# Understanding the Phytoremediation Mechanisms of Potentially Toxic Elements: A Proteomic Overview of Recent Advances

**DOI:** 10.3389/fpls.2022.881242

**Published:** 2022-05-06

**Authors:** Mohammed Alsafran, Kamal Usman, Bilal Ahmed, Muhammad Rizwan, Muhammad Hamzah Saleem, Hareb Al Jabri

**Affiliations:** ^1^Agricultural Research Station (ARS), Office of VP for Research and Graduate Studies, Qatar University, Doha, Qatar; ^2^Central Laboratories Unit (CLU), Office of VP for Research and Graduate Studies, Qatar University, Doha, Qatar; ^3^School of Chemical Engineering, Yeungnam University, Gyeongsan, South Korea; ^4^Office of Academic Research, Office of VP for Research and Graduate Studies, Qatar University, Doha, Qatar; ^5^Center for Sustainable Development (CSD), College of Arts and Sciences, Qatar University, Doha, Qatar; ^6^Department of Biological and Environmental Sciences, College of Arts and Sciences, Qatar University, Doha, Qatar

**Keywords:** plants, proteomics, phytoremediation, toxic metals, pollution

## Abstract

Potentially toxic elements (PTEs) such as cadmium (Cd), lead (Pb), chromium (Cr), and arsenic (As), polluting the environment, pose a significant risk and cause a wide array of adverse changes in plant physiology. Above threshold accumulation of PTEs is alarming which makes them prone to ascend along the food chain, making their environmental prevention a critical intervention. On a global scale, current initiatives to remove the PTEs are costly and might lead to more pollution. An emerging technology that may help in the removal of PTEs is phytoremediation. Compared to traditional methods, phytoremediation is eco-friendly and less expensive. While many studies have reported several plants with high PTEs tolerance, uptake, and then storage capacity in their roots, stem, and leaves. However, the wide application of such a promising strategy still needs to be achieved, partly due to a poor understanding of the molecular mechanism at the proteome level controlling the phytoremediation process to optimize the plant’s performance. The present study aims to discuss the detailed mechanism and proteomic response, which play pivotal roles in the uptake of PTEs from the environment into the plant’s body, then scavenge/detoxify, and finally bioaccumulate the PTEs in different plant organs. In this review, the following aspects are highlighted as: (i) PTE’s stress and phytoremediation strategies adopted by plants and (ii) PTEs induced expressional changes in the plant proteome more specifically with arsenic, cadmium, copper, chromium, mercury, and lead with models describing the metal uptake and plant proteome response. Recently, interest in the comparative proteomics study of plants exposed to PTEs toxicity results in appreciable progress in this area. This article overviews the proteomics approach to elucidate the mechanisms underlying plant’s PTEs tolerance and bioaccumulation for optimized phytoremediation of polluted environments.

## Introduction

The continued accumulation of potentially toxic elements (PTEs), including cadmium (Cd), lead (Pb), chromium (Cr) and arsenic (As), copper (Cu), mercury (Hg), nickel (Ni), and selenium (Se) in the environment poses a significant danger to human health and undermines global environmental sustainability efforts (Habiba et al., 2019; Rizvi et al., 2019, 2020; Alsafran et al., 2021). Anthropogenic activities due to rapid industrialization, especially from oil and gas producing industries, infrastructural development, mining, foundries, smelters, coal-burning power plants, and agricultural activities, are significant contributors that enhance the elements of hazardous pollutants in the soil (Al-Thani and Yasseen, 2020; Yan et al., 2020; Usman et al., 2020b). While this is the case, bioaccumulation strategies and tolerance to higher concentrations of PTEs, thus sequestering of PTEs can be varied among different plant species as they faced diverse pollutant sources and other environmental conditions. Generally, PTEs sequestration mechanisms in plant tissues include exclusion, stabilization, removal, and transfer to the various parts such as roots, shoots, and stems. Of these, the removal and translocation of the elements to plant’s aerial parts, the process also known as “phytoextraction,” are known as the most efficient remediation strategy (Saleem et al., 2020a). Phytoextraction is inexpensive, the amount of waste material that must be disposed of is substantially decreased (up to 95%), and the disposal of hazardous material or biomass is not required (Usman et al., 2020b). Plant species demonstrating the capacity to remove and transfer PTEs to their aerials parts are categorized as metal hyperaccumulators (De Bellis and Aprile, 2020; Zhang et al., 2020; Usman et al., 2020b).

The PTEs are non-biodegradable and prone to ascend along food chains, making their environmental prevention a critical intervention (Wuana and Okieimen, 2011; Sharma and Pandey, 2014). Given the potential adverse effects of many remediation strategies, alternative technologies, including phytoremediation—the use of plants to remove PTEs from contaminated environments, are being explored for large-scale applications (Usman et al., 2019b). Phytoremediation is the direct use of living green plants and is an effective, cheap, non-invasive, and environmentally friendly technique used to transfer or stabilize all the toxic metals and environmental pollutants in polluted soil or ground water (Mosa et al., 2016). Phytoremediation is widely applicable for metal contaminated areas, with some long-term esthetic merits and it is famous due to its low cost and eco-friendly nature, so it is used on large-scale areas with high contents of toxics metals (Rascio and Navari-Izzo, 2011). Plants are sessile organisms, and therefore, could not escape from exposure to high concentrations of PTEs (Wiszniewska, 2021). However, several plant species (⁓450) are known to accumulate high concentrations of various PTEs (Rascio and Navari-Izzo, 2011). PTEs mainly enter plant systems from soil or water *via* passive or active transport. Following uptake that is facilitated by membrane-embedded ion channels, elemental ions translocate to aerial parts of plants (i.e., the stem and leaves) *via* xylem channels. In general, plants capable of accumulating PTEs in their tissues majorly bio-concentrate the elements in the root, followed by the stem, leaves, and in some species, even the seeds (Shamim, 2018; Dinu et al., 2020).

Biotechnologically, three main strategies are embarked upon to improve PTEs phytoextraction using different plants species: (i) utilizing the metal/metalloid transporters, (ii) enhancing metal/metalloid ligand production, and (iii) conversion of metal/metalloid into volatile and less detrimental forms (Mosa et al., 2016). The toxicity of PTEs primarily depends on various factors such as concentrations and chemical properties of toxic elements, their bioavailability, and plants’ developmental stage. When exposed to PTEs, plant’s basal tolerance mechanism becomes activated and enables them to cope with the stress (Gill et al., 2021). However, at elevated concentrations, these elements suppress the plant defense machinery and cause harmful effects to physiological processes, including photosynthesis, transpiration, and energy metabolism, thus reducing overall plant growth and development (Kumar et al., 2018; Gautam et al., 2020; Ahmad et al., 2020a; Usman et al., 2020a). Generally, PTEs stress symptoms on plants can be measured as it is similar to that of deficiency in essential nutrients that may be appeared in the forms of leaf necrosis, poor root development, and decreased fresh biomass (Usman et al., 2019a; Singh and Fulzele, 2021).

Recently, the “Omics” approaches emerge as valuable tools for understanding the changes in molecular mechanisms of plant’s response to the PTEs during phytoremediation (Meena et al., 2017; Raza et al., 2021). The traditional characterization methods relating to physiological and biochemical assays seem insufficient, and therefore, further investigation especially on the response of whole-genome proteome to PTE can be a promising approach to coping with the potential threats posed by PTEs (Xie et al., 2019; Kosakivska et al., 2021). These changes are not only limited to the expression pattern but also protein quality and quantity. Transcriptomic approaches are used to target transcriptional changes at the mRNA level (i.e., changes in gene expression), which may differ from changes at the protein level (i.e., translational modifications). In a true sense, the mRNA/protein ratio is a factor of mRNA transcription rate and protein stability (Reimegård et al., 2021).

To alleviate PTEs stress and restore cellular homeostasis, plants develop antioxidative capacity, sophisticated and highly efficient regulatory mechanisms to help tolerate the uptake, accumulation, translocation, and eventual detoxification (El-Amier et al., 2019; Alsahli et al., 2020; Ahmad et al., 2020b; Bhat et al., 2021). To achieve this, the living system’s functional molecules, the proteins, particularly metal chelators, transporters, and chaperones, play crucial roles in alleviating the negative impact of PTEs stress (Saleem et al., 2020b). Together, these proteins enable plants to tolerate PTEs, detoxify PTEs polluted environments and their system through binding, transport, and vacuolar sequestration (Peco et al., 2020; Dhir, 2021; Jogawat et al., 2021).

Proteins are crucial to regulating the cellular processes of plants; proteomics, comprising cellular protein roles, quantification, identification, the pattern of expression, modification, and interactions, all together provides an excellent strategy to assess stress impact on them. Because of the central roles of proteins, researchers in this area need to prioritize studies focusing on proteomics to gain further insights into the mechanisms of PTEs tolerance and detoxification in plants to improve the efficiency of PT Es removal from contaminated soil or medium.

**Figure 1:**
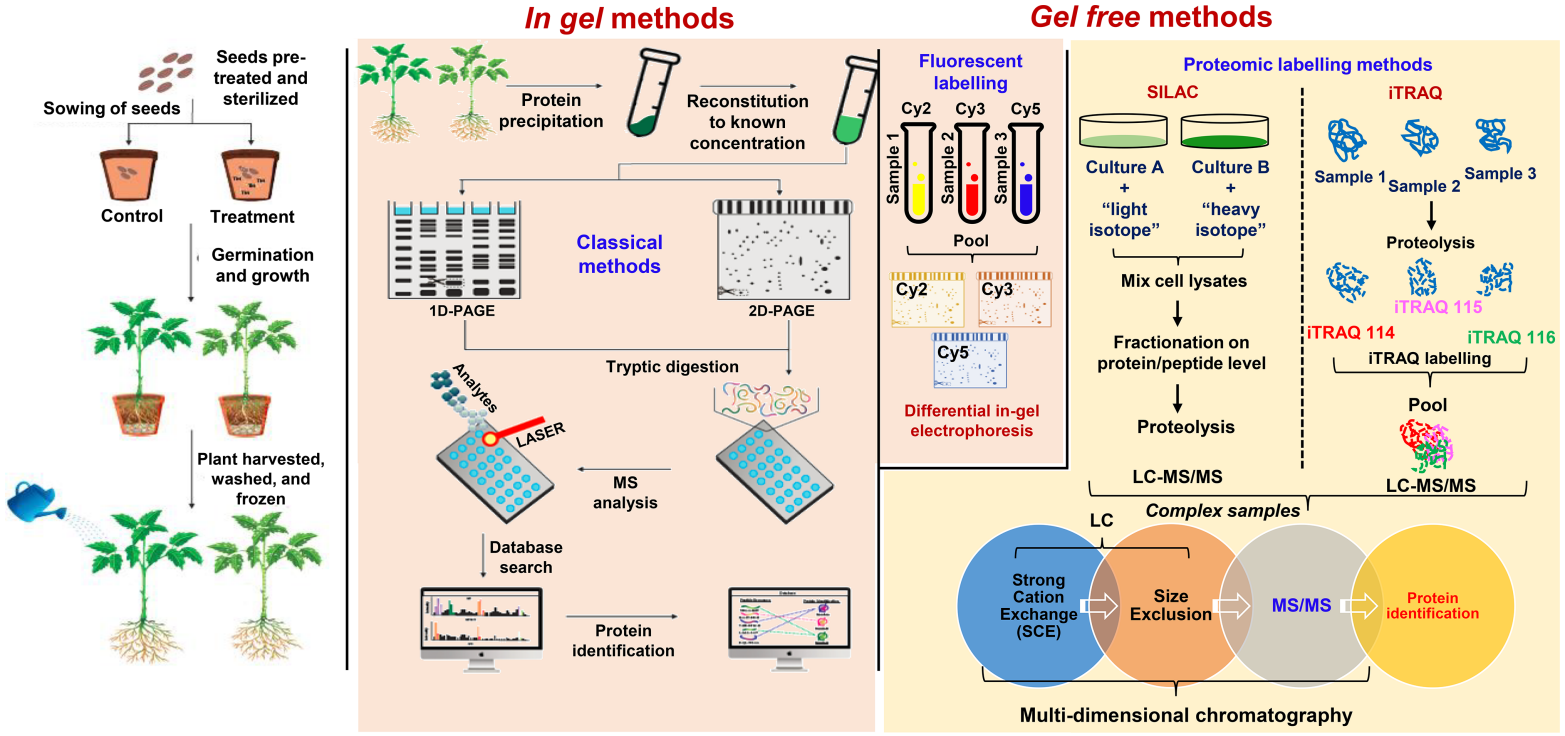
A workflow illustrating the comparative proteomic methods that have been used to investigate the phytoremediation of potentially toxic elements. Classical in-gel proteomic methods include 1-D and 2-D polyacrylamide gel electrophoresis (PAGE) sometimes further developed by differential in-gel electrophoresis (DIGE) using fluorescent tags like cyanine 2 or 3 or 5 (Cy2, Cy3, and Cy5). Gel-free methods are advanced and used to overcome limitations in-gel proteomics and to study the heavy metal detoxification and phytoremediation mechanisms. These include novel gel-free methods with protein labeling such as Stable Isotope Labeling with Amino acids in Cell culture (SILAC) and Isobaric Tags for Relative and Absolute Quantitation (iTRAQ) techniques followed by multi-dimensional chromatography (MupPit).

Recent progress in plant proteomics could be possible due to new technological advancements in protein separation, quantification, mass spectrometry (MS), and bioinformatics. Mass spectroscopy is central to large-scale proteome analysis that enhances the resolution, sensitivity, and accuracy of proteins mass prediction (Cassidy et al., 2021). Due to these and the speed of analysis for large protein samples through released peptides after proteolytic digestion (bottom-up), shotgun proteomics is used to describe the process (Gutsch et al., 2019b). On the other hand, protein is partially digested to characterize co-existing post-translational modifications (PTMs; Sidoli et al., 2017). Following fractionation and tandem mass spectrometry (MS/MS) analysis, the bottom-up process indirectly measures proteins through tryptic digested peptides having amino acids approximately between 8 and 30 (8 > aa>30). Proteins are inferred through identified peptides compared to MS/MS spectra previously generated from in-silico fragmented peptides in a protein database. Figure 1 shows a schematic representation of typical steps in PTEs phytoremediation studies involving the use of shotgun proteomics.

**Figure 2:**
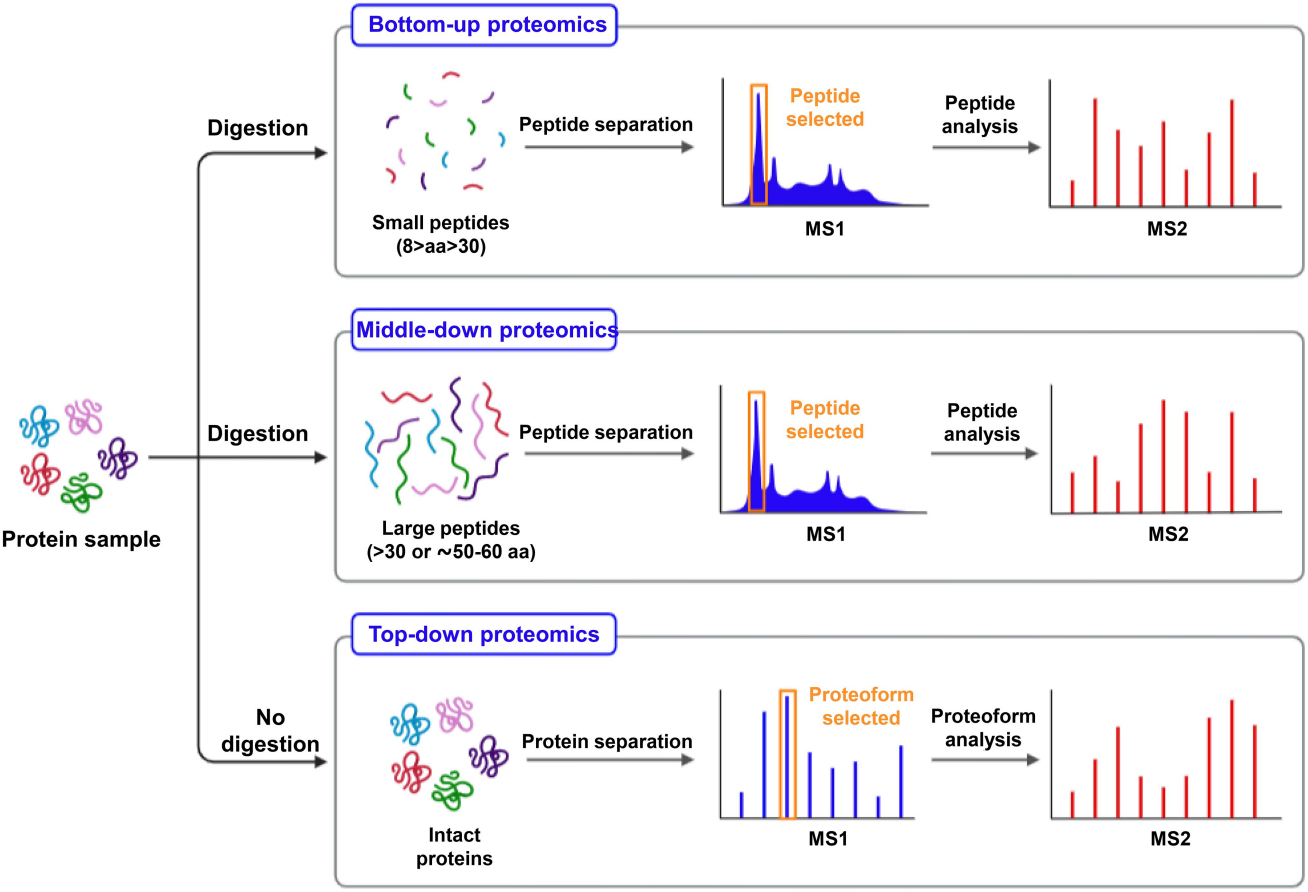
A proposed workflow for protein digestion and MS analysis for the investigation of large (intact proteins), medium (peptides >30 aa), and small size (8–30 aa) protein molecules.

In contrast to the bottom-up approach (analysis of digested proteins), the proteomics of characterizing intact proteins is another strategy termed “top-down” (Figure 2). Proteomic research has made significant progress, especially on model plants, *Oryza sativa*, and *Arabidopsis thaliana*. Essential proteins, such as metal ion transporters, binding proteins, phytochelatins (PCs), and metallothioneins (MTs), are notable in aiding PTEs sequestration in plants. PCs are induced by phytochelatins synthase (PCS), which is triggered when metal ions are present. PCs (oligomers of glutathione) bind to toxic metals to form a significant part of the detoxification mechanism, while MTs are gene-encoded, small, and cysteine-rich proteins (Jorrin-Novo et al., 2019; Usman et al., 2020b).

Due to the exponential increase in the number of studies and publications in the proteomics of plant abiotic stress, including PTEs, it is almost impossible to provide an extensive summary in one review. For further references within the last decade, readers are referred to some of the recent reviews (Ahsan et al., 2009; Hossain and Komatsu, 2013; Cvjetko et al., 2014; Kosová et al., 2018; Raza et al., 2020; Kosakivska et al., 2021; Sytar et al., 2021). This review aims to provide a non-exhaustive overview of plant proteomics and highlights its importance in understanding PTEs tolerance, uptake, and detoxification mechanisms in plants during phytoremediation when grown in metal contaminated soil. To the best of our knowledge, this review is among the few articles focused on the plant proteomics of trace and heavy metals.

## Phytoremediation

**Figure 3:**
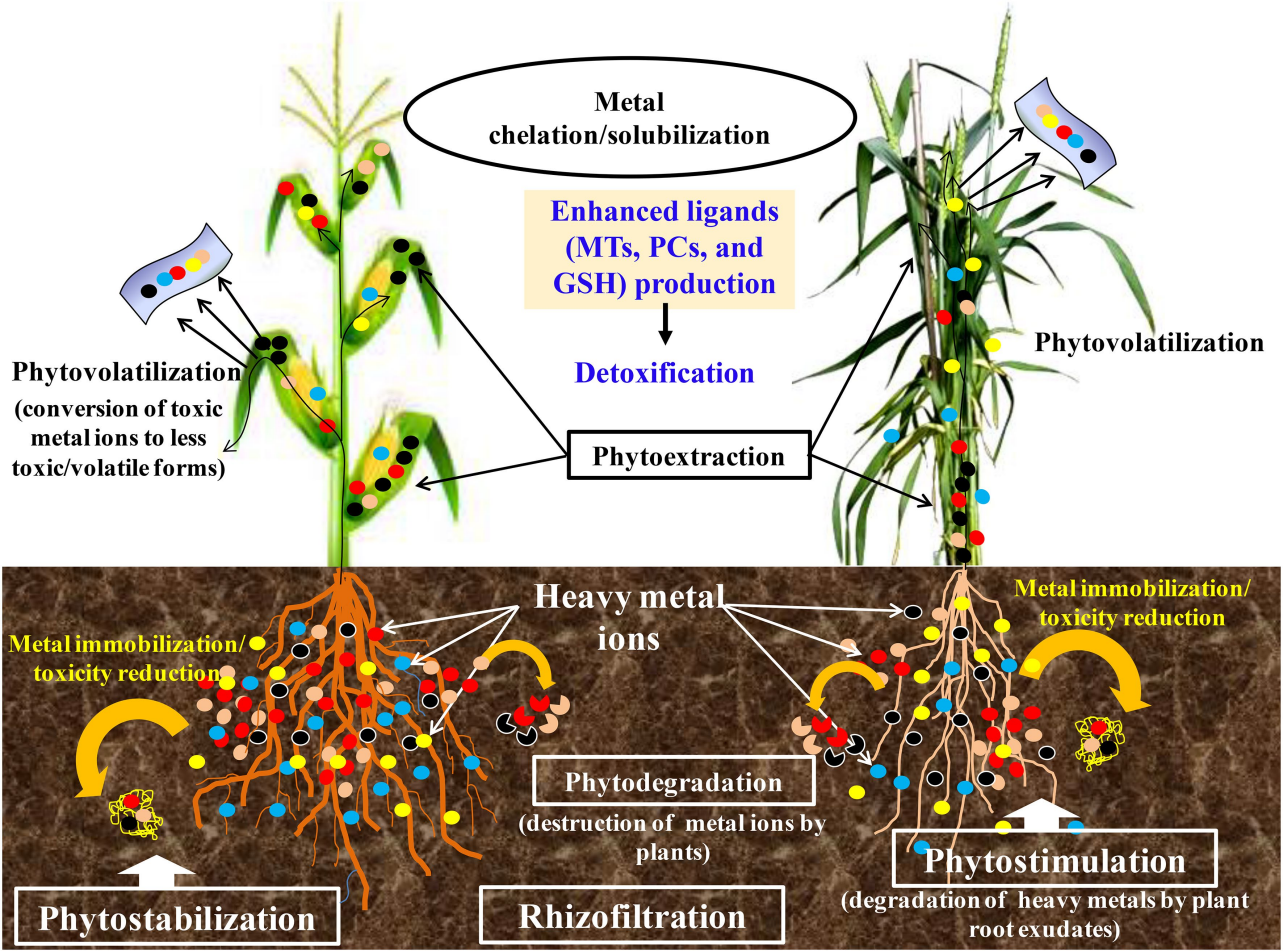
An illustration on the phytoremediation strategies and general response of plants to potentially toxic elements stress.

A combinatorial strategy involved the physiological and chemical properties, and biological processes adopted by plant species to clean up environmental pollutants (Baldwin et al., 2015; Hasegawa et al., 2016). Physical and chemical methods have several limitations such as non-economical, alterations in native soil flora, changes in the physicochemical properties of the soil, and need intensive labor (Shankar, 2017). PTEs are essentially immutable by any chemical or physical process short of nuclear fission and fusion, and thus, their remediation presents special scientific and technical problems. Because of this, new approaches for better treatment of PTEs polluted environment are essential. In this regard, the use of biological treatment strategies could be adopted that are cheaper and environmentally friendly. The promising one is phytoremediation which has gained increased attention in recent years since it is the most viable alternative. Phytoremediation takes advantage of plant ability to tolerate, accumulate, and translocate PTEs across their aerial tissues (Ludvíková and Griga, 2019). Phytoremediation is often referred to as “green remediation” or “botanical bioremediation” involving the use of plants to remove, transfer, or stabilize the PTEs (Figure 3) to clean up the environment and render the pollutants harmless (Suman et al., 2018; Adiloğlu et al., 2021). Moreover, this mechanism is a species-specific, effective, economical, eco-friendly, and scientifically accepted method. Generally, when there is an encounter with PTEs, plants activate their defense machinery by adopting one or several mechanisms simultaneously to safeguard themselves from unwanted physiological or molecular alterations induced by PTEs. Some of the most studied and common strategies are presented in Table 1.

**Table 1:** Phytoremediation strategies adopted in response to PTEs.

S. No.	Strategies	Description	Crop	Family	References
1.	Phytoextraction	A low-cost technique by which PTEs are removed or concentrated by plants in different parts. This process produces plant biomass having PTEs that can be transported for disposal or recycling	*Calotropis procera*	Dogbanes	Singh and Fulzele, 2021
2.	Phytodegradation or Rhizodegradation	PTEs are degraded by proteins or enzymes produced by plants and associated microbes	*Phragmites australis*	Grasses	He et al., 2017
3.	Rhizofiltration	PTEs are absorbed by plant roots	*Carex pendula*	Sedges	Yadav et al., 2011
4.	Phytostabilization	PTEs are immobilized, and thus their bioavailability is reduced	*Juncus effusus*	Rushes	Najeeb et al., 2017
5.	Phytovolatization	Volatilization of PTEs by plants extracted from soils into the atmosphere	*Pteris vittata*	Brake	Sakakibara et al., 2010

Phytoremediation has a great potential for providing much-needed green technology. During phytoremediation, the plant’s selection for the remediation strategy to neutralize PTEs may differ; the strategies used could be removal, transfer, degradation, immobilization, etc. (Hasanuzzaman et al., 2018). While hundreds of plant species have been identified as potential phytoremediators, identifying suitable hyperaccumulators is still a challenge (de Castro Ribeiro et al., 2018). Previously, Yıldız and Terzi (2016) studied *Brassica napus* under Cr stress and noticed through 58 proteins spots in two-dimensional electrophoresis (2-DE) that 58 proteins were differentially regulated by Cr (VI) stress (+S/+Cr), S-deficiency (−S/−Cr), and combined stress (−S/+Cr). The translocation capacity of plants (the ability to take up and accumulate toxic metal from the root to shoot parts) is a critical parameter considered in evaluating phytoremediation potential in plants (Meng et al., 2017). A translocation factor of 1 or more suggests a plant’s ability to transfer metals to its aerial parts adequately. Various methods covered under phytoremediation strategies are discussed briefly in the following sections.

### Phytoremediation Strategies

Plants employ different strategies during phytoremediation. The type of elements, their chemical properties, and bioavailability plays a crucial role in achieving PTEs remediation success (Usman et al., 2020b). The different phytoremediation strategies include phytoextraction, phytofiltration or Rhizofiltration, phytovolatilization, phytostabilization, and phytodegradation (Figure 3; Usman et al., 2018; Wei et al., 2021). Phytoextraction involves the use of plants to remove PTEs or organics from the soil by concentrating them in the harvestable parts (Kumar et al., 2017; Ali et al., 2018). PTEs accumulating plants are utilized to transport and concentrate contaminants (metals or organics) from the soil into the above-ground plant parts as shown in the example by Viana et al. (2021). Phytoextraction involves PTEs removal, accumulation, and translocation to plant’s aerial parts (Viana et al., 2021). Often, phytosequestration, photoabsorption, and phytoaccumulation are used to refer to the same process. Several studies have reported plants demonstrating PTEs phytoextraction capacity. Phytoextraction is preferred over other techniques because toxic elements can be harvested from plant shoots in an extractable form (Jeyasundar et al., 2021). Several studies have reported different plants with varying capacities in the phytoextraction of PTEs. Examples are the Indian mustard, rapeseed, and sunflower plants (Shaheen and Rinklebe, 2015; Chowdhary et al., 2018; Surucu et al., 2020).

#### Phytofiltration or Rhizofiltration

Phytofiltration or Rhizofiltration involves the adsorption of PTEs *via* the root. It is a process most seen in aquatic plants (Meitei and Prasad, 2021). In rhizofiltration, plant roots are used to absorb and adsorb pollutants, mainly metals, from contaminated soils and aqueous waste streams. It is the removal of pollutants from metal-polluted soil/waters by precipitation, absorption, and accumulation into plant biomass (Mahajan and Kaushal, 2018). Phytofiltration is essential because it prevents toxic elements transmission to different environmental components, including underground water (da Conceição Gomes et al., 2016; Meitei and Prasad, 2021). However, phytofiltration is also demonstrated by terrestrial species, where metals are remediated with microbial bio-filter aid in the rhizosphere region (Wei et al., 2020). Previously, studies conducted on rhizofiltration by Yadav et al. (2011) in *Carex pendula* in Pb contaminated wastewater soil noticed that *C. pendula* accumulate a large amount of Pb in their roots and can be used to clean up the Pb contaminated environment in combination with proper biomass disposal alternatives.

#### Phytostabilization

Plants can reduce PTEs toxicity by converting them to a different form or changing their bioavailability. Thus, the bioavailability of PTEs in the environment is reduced using plant systems. Plants stabilize PTEs in soils, thus rendering them harmless, thereby reducing the risk of further environmental degradation by leaching of PTEs into the groundwater or by airborne spread. This is achieved by preventing surface runoff, erosion, and leaching (Yan et al., 2020). Phytostabilization is vital because it helps prevent PTEs transmission into the food chain. The element’s chemical properties are some of the most critical determinants of whether potential plants can stabilize them (Hamidpour et al., 2020; Usman et al., 2020b). Although phytostabilization offers some advantages, it has limited use because metals are only temporarily immobilized and restricted, and therefore, unpopular compared to phytoextraction (Radziemska, 2018). It is commonly employed in emergencies for quick metal immobilization in plants’ rhizosphere (Meng et al., 2017).

#### Phytotransformation or Phytodegradation

It is like phytostabilization, but pollutants are metabolically transformed into inactive forms (Bezie et al., 2021). The plant metabolic system employs the surrounding enzyme activities with the assistance of rhizosphere bacteria to reduce metal elements toxicity. Compared to other forms, phytotransformation is labor-intensive, often requires soil amendments, and is less reliable (Mishra et al., 2020; Bezie et al., 2021). Phytodegradation is commonly applicable against organic pollutants. However, it is less effective and rarely used, especially against inorganic contaminants, including PTEs.

#### Phytovolatilization

It involves converting metal contaminants into a gaseous form that is eventually released into the atmosphere (Aweng et al., 2018). In this process, PTEs are only transferred to other parts of the environment and could still be redeposited into the soil following precipitation. For this reason, phytovolatilization is less popular, especially compared to phytoextraction and phytofiltration (Nikolić and Stevović, 2015; Bisht et al., 2020).

## The Mechanisms of PTEs Tolerance and Bioaccumulation

Plants with the enhanced potential of taking up PTEs and translocating them to their aerial parts are identified as metal hyperaccumulators, while those with limited metal translocation are known as non-hyperaccumulators (Maestri et al., 2010). Recently, the interest in proteomics studies of plant hyperaccumulators and their metal sequestration and detoxification mechanisms has increased (Visioli and Marmiroli, 2013; Kumar and Prasad, 2018; Raza et al., 2020). Proteomic studies of PTEs accumulators can make comparisons of differentially expressed proteins (DEPs) between different plant parts (Table 2). Many studies suggest that the hyperaccumulators including transporters and chelators showed enhanced DEPs compared to non-accumulating plants (Visioli and Marmiroli, 2013; Paape et al., 2016; Domka et al., 2020). During PTEs phytoremediation, plant tissues play essential roles.

**Table 2:** Examples of PTEs phytoremediation studies involving the use of comparative proteomics from 2015 to date.

PTEs	Plant species	Plant parts	PTEs concentration/exposure time/media	Technology used	Key findings	References
As	*Artemisia annua* L.	Shoot Root	100 μm Na_2_HAsO_4_.7H_2_O/3 days Hoagland nutrient’s solution	2-DE PAGE, MALDI-TOF-MS	Upregulation of secondary metabolites-related genes enhances as tolerance. Biomass, carotenoid, flavonoids were enhanced, whereas total chlorophyll pigment was reduced under As treatment.	Kumari and Pandey-Rai, 2018
	*Brassica napus*	Leaves	200 μmoll−1 NaAsO_2_/ 7 days/ 50% Hoagland solution	LC–MS/MS, SEM, TOF-MS, qRT-PCR	Photosystem II (PSII) and photosystem I (PSI) proteins were upregulated. Secondary metabolites biosynthesis increased.	Farooq et al., 2021
	*Oryza sativa* L.	Leaves Root	NaAsO_2_; 25 μM /7 d/ modified Hewitt’s media	2-DE, MALDI-TOF-TOF	The sulfur treatment alleviates As stress by forming disulfide linkage in proteins involved in glycolysis, TCA cycle, energy metabolism, and photosynthesis.	Dixit et al., 2015
	*Populus* (deltoides cv. “zhonglin 2025” and euramericana cv. ‘I-45/51’)	Leaves Root	Na_3_AsO_4_·12H_2_O 50, 100 μM/21 days/Hoagland’s nutrient solution	MALDI-TOF/TOF MS, 2-DE, RT-PCR	Overexpression of photosynthetic and antioxidative responsive proteins in As tolerant cultivar	Liu et al., 2017
Cd	*Arabidopsis thaliana* L.	Leaves, Root	100 μm CdCl_2_/7-days/1/2 MS solid media	2D-GE, MALDI-TOF/TOF-MS	The natural accession Chernobyl-07 (Che) has a higher Cd tolerance than normal accessions. This accession particularly changed the expression related to ROS protection and energy modulation proteins for obtaining tolerance.	Klimenko et al., 2019
	*Brassica campestris*	Root	50 μm CdCl2/1-day/ hydroponic	2D-GE, MALDI-TOF/TOF-MS	Hydrogen gas (H_2_) and nitric oxide (NO) enhance the antioxidant capabilities of *B. campestris* seedlings in response to Cd toxicity.	Su et al., 2019
	*Brassica napus*	Xylem sap	10 μm CdCl_2_/3-days/hydroponic	LC–MS/MS	Cd stress-induced the overexpression of stress response-related proteins.	Luo and Zhang, 2019
	*Medicago sativa*	Stem	88.9 μm CdSO_4_/4-months/potted soil	2D-GE, MALDI-TOF/TOF-MS	Cd stress caused the differential expression of proteins involved in cell wall remodeling, defense response, carbohydrate metabolism, and promotion of the lignification process.	Gutsch et al., 2019a
	*Microsorum pteropus*	Leaves, Root	100, 250 and 500 μm CdCl_2_/7-days/hydroponic	2D-GE, MALDI-TOF/TOF-MS	Different protein expression patterns were observed involving related functions of energy metabolism and antioxidant activity in the root, cellular metabolism, protein metabolism, and photosynthesis in leaves.	Lan et al., 2018
	*Sorghum bicolor*	Shoot	100 and 150 μm CdCl_2_/5-days/semi hydroponic	2D-GE, MALDI-TOF/TOF-MS	Cd stress inhibits carbon fixation, ATP production, and the regulation of protein synthesis.	Roy et al., 2016
Cr	*Brassica napus* L.	Leaves	100 μm K_2_Cr_2_O_7_/3-days/hydroponics	2-DE, MALDI-TOF/TOF MS	Increased abundance of defense-related proteins such as antioxidant enzymes, molecular chaperones involved in scavenging the excess ROS, and refolding of misfolded proteins under Cr stress.	Yıldız and Terzi, 2016
	*Callitriche cophocarpa*	Shoot	1 mm K_2_CrO_4_/3-days/liquid MS medium	SDS-PAGE, 2DE, MS/MS	Quinone dehydrogenase, FQR1 (NAD(P)H) newly identified to act as a detoxification protein by protecting the cells against oxidative damage.	Kaszycki et al., 2018
	*Nicotiana tabacum*	Shoot	100 μm K_2_Cr_2_O_7_/5-days/hydroponic	2D-GE, MALDI-TOF/TOF-MS	Twelve Cr-tolerance-associated proteins were identified. These include mitochondrial processing peptidase, dehydrin, superoxide dismutase, adenine phosphoribosyltransferase, and mitochondrial malate dehydrogenase proteins.	Bukhari et al., 2016
	*Pteris alba*	Leaves Root	146.7 ~ 261.5 mm Cr/4-years/waste landfill field	2D-GE, Nano HPLC MS/MS	ROS scavenging proteins assist poplar threes long-term adaptation to Cr polluted environments.	Szuba and Lorenc-Plucińska, 2018
Cu	*Agrostis capillaris* L.	Shoot	1–50 μm CuSO_4_/90-days/semi hydroponic	2D-GE, LC–MS/MS	Overexpression of a Heat shock protein 70 (HSP70) may be pivotal for Cu tolerance by protecting protein metabolism.	Hego et al., 2016
	*Hyoscyamus albus* L.	Root	0, 0.1, 1, 20, and 200 μm CuSO_4_/7-days/cell culture	MALDI-QIT-TOF-MS	High Cu levels enhanced respiration activity and propagated *H. albus* roots through the activation of the energy supply and anabolism. Increased abundance of proteins involved in carbohydrate metabolism, *de novo* protein synthesis, cell division, and ATP synthesis, and decreased proteasome.	Sako et al., 2016
	*Triticum aestivum* L.	Root Leaves	100 μm CuSO_4_/3-days/hydroponic	2D-GE, HPLC-Chip	Cu responsive network of 36 key proteins, most of which may be regulated by abscisic acid (ABA), ethylene, and jasmonic acid (JA). Exogenous JA application showed a protective effect against Cu stress and significantly increased glutathione S-transferase (GST) gene transcripts.	Li et al., 2013
Hg	*Paspalum distichum* L.	Root	1,115 μm Hg/ 60days/contaminated soil in glass box	LC–MS/MS	Observed changes in the expression patterns of metal binding and transport protein. Increased accumulation of photosynthesis and energy metabolism, related proteins.	Ding et al., 2019
	*Triticum aestivum* L.	Root Shoot	25, 50, 100, 200 and 400 μm HgCl_2_/3-days /hydroponic	2D-GE, LC–MS/MS	49 abscisic acid (ABA) potentially regulated Hg-responsive proteins identified. Exogenous ABA application conferred protection against Hg stress and increased peroxidase enzyme activities, suggesting that it may be an important factor in the Hg signaling pathway.	Kang et al., 2015
Pb	*Cannabis sativa* L.	Leaves	Pb(NO_3_)_2_ 3 g/kg soil /40-days/Potted soil	LC-ESI-MS/MS. SWATH-MS	Adaptation to Pb stress by accelerating adenosine triphosphate (ATP) metabolism; enhancing respiration, light absorption, and light energy transfer; and eliminating reactive oxygen species.	Xia et al., 2019
	*Chrysopogon zizanioides*	Root Shoot	Pb(NO_3_)_2_ 400 mg/l, 800 mg/l and 1,200 mg/l/10-days/hydroponic (half strength Hoagland solution)	LC–MS/MS	Increased levels of key metabolites including amino acids, organic acids, and coenzymes in response to Pb.	Pidatala et al., 2018
	*Raphanus sativus* L.	Root	1,000 mg/ L Pb(NO_3_)_2_/3-days/modified half-strength Hoagland nutrient solution	GC–MS	Pb exposure altered metabolites and divergent expression of enzymes which are responsible for profound biochemical changes, including carbohydrate metabolism, energy metabolism, and glutathione metabolism.	Pang et al., 2015
	*Glycine max* L.	Nodules	107.8 μm PbCl_2_ or 1.84 μm HgCl_2_/ 60-days /potted peat, perlite, and vermiculite (1:1:1)	2D-GE, MALDI-TOF MS/MS	Pb stress increased the abundance of defense, development, and repair-related proteins.	Baig et al., 2018
	*Zea mays*	Root	18,000 μm Pb (NO3)_2_/12, 24 and 48 h/semi hydroponic	Nano-LC–MS/MS	Upregulation of stress, redox, signaling, and transport proteins, while proteins related to nucleotide metabolism, amino acid metabolism, RNA, and protein metabolism were down-regulated.	Li et al., 2016
Se	*Allium cepa* L.	Root	10 mg/l Se Na_2_SeO_3_/10-days/Hoagland’s nutrient solution	Cap HPLC-ESI-QTOF-MS and MS/MS, nano LC-ESI-Q Orbitrap-MS and MS/MS	Different abundances of proteins involved in transcriptional regulation, protein folding/ assembly, cell cycle, energy/carbohydrate metabolism, stress response, and antioxidant defense were identified in response to Se stress.	Karasinski et al., 2017
	*Brassica oleracea* L.	Florets Leaves	25 μm Na_2_SeO_4/_14-days/Hoagland solution	UPLC–MS/MS, qRT-PCR, LC–MS/MS	Glucosinolate reduction in broccoli leaves and florets is associated with negative effects on precursor amino acids (methionine and phenylalanine), biosynthesis, and glucosinolate-biosynthetic-gene expression in response to Se supplementation.	Tian et al., 2018
	*Capsicum annuum* L.	Shoot	100 ppm Na_2_SeO_4_/1-day	LC–MS/MS	Overexpression of heat shock and metabolism proteins. Others are involved in post-translational modification, protein turnover, chaperones, and protein processing in the endoplasmic reticulum.	Zhang et al., 2019
	*Oryza sativa* L.	Shoot Root	25 μM, NaAsO_2_ and 25 μm Na_2_SeO_3_/15-days/Hewitt nutrient medium	MALDI-TOF/TOF, qRT-PCR, Western blot,	Differentially expressed proteins altered the gene expression related to abiotic and biotic stresses and defense responses such as ROS homeostasis, photosynthesis, energy metabolism, and transport and signaling.	Chauhan et al., 2020

The root is the first tissue to encounter metal stress, and therefore often witnesses dramatic proteomic changes. When comparing the root protein of two accessions, glycosyl hydrolase family 18 differed in abundance, affecting the plant’s capacity to uptake metal; the variant that had a higher protein abundance had higher Ni and Cd accumulation (Lai et al., 2020; Raza et al., 2020). The proteome of a variety of plant species was studied, and several proteins that protect plants against various stresses, including oxidative, biotic, and abiotic stress conditions were identified (Fan et al., 2016; Goodin, 2018; Kumar et al., 2018). When comparing *Thlaspi caerulescens* proteomes that had variable tolerance to Cd and Zn, it was determined that the element’s higher accumulation was due to the protein photosystem II (Paunov et al., 2018). Proteomic analysis of *Sorghum bicolor* has also shown that a total of 33 DEPs were found when plants were (Table 2) exposed to cadmium (Cd) stress (Roy et al., 2016). Examples of such proteins are glutathione S-transferase, ribulose bisphosphate carboxylase small chain, carbonic anhydrase, glyceraldehyde-3-phosphate dehydrogenase, and cytochrome P450, which are well characterized this far in historical literature. The less characterized contenders that were upregulated in *S. bicolor* include pentatricopeptide repeat-containing protein, Zn finger CCCH domain-containing protein 14, flavonoid 3′,-5′ hydroxylase, aspartate aminotransferase 3 (chloroplastic), protein Brevis radix-like 1, bergaptol O-methyltranferase, and probable F-actin-capping protein subunit beta proteins under Cd stress (Roy et al., 2016). Physiologically, in *S. bicolor* plants, there is the suppression of carbon fixation, ATP production, and protein synthesis regulation in Cd-stressed plants (Roy et al., 2016). In fact, under 500 μm Cd stress, the fern *Microsorum pteropus* is capable of sequestering high amounts of cadmium in roots and dry matter of leaves (up to 4,000 mg/kg), while the water fern Azolla, widely seen in Asian rice fields, does not have the same capacity to phytoaccumulate Cd.

In a study performed on hemp cultivars (Xia et al., 2019), it was found that phytoremediation of Pb impacts the following key pathways: protein synthesis, transcription, transport, signal transduction, photosynthesis, energy metabolism, and protein storage, among other systems. Examples of proteins that are upregulated in Y1 cultivars of hemp include ones that optimize ATP generation using ATP synthase subunit a (P56758 and P56757), ATP synthase protein MI25 (Q04613), ATP synthase protein YMF19 (P93303), nucleoside diphosphate kinase III (O49203), pyruvate kinase (PKE; Q94KE3, Q9FNN1, and Q9FM97), and adenylate kinase 5 (ADK; Q8VYL1; Xia et al., 2019). Therefore, making more chemical energy appears to be a favorable development when exposed to high Pb stress. In particular, the pyruvate kinase that mediates pyruvate production for the Kreb’s Cycle is a key protein that is upregulated. In the same cultivar (Y1) under Pb stress, the following proteins were upregulated for signal transduction and transport: Five water transport-related aquaporins (e.g., Q06611, P25818, and others), patellin (Q56ZI2 and Q56Z59), mitochondrial dicarboxylate/tricarboxylate transporter DTC (Q9C5M0), mitochondrial phosphate carrier protein 3 (Q9FMU6), mitochondrial carnitine/acylcarnitine carrier-like protein (Q93XM7), MD-2-related lipid recognition domain-containing protein/ML domain-containing protein (F4J7G5), and ras-related protein RABA2a (O04486; Xia et al., 2019).

Aquaporins on the contemporary are not seen solely as water transporters but can transport ammonia, boron, carbon dioxide, silicon, urea, and even PTEs such as As (Mosa et al., 2016). An Aqua1 gene from *Populus trichocarpa*, which has a very high number of aquaporins in its proteome, when expressed in a Zn-sensitive strain of yeast, was able to confer Zn-resistance. Furthermore, Aqua1 protein product was observed to co-localize with AtTIP1, a well-known *Arabidopsis* vacuolar marker (Ariani et al., 2019). The contenders for phytoremediation that are DEPs come in large datasets that it is difficult to describe in detail covering all proteins in one review article. There were 63 and 372 differently expressed proteins (≥1.5) in the tolerant (BM) and susceptible (Y1) cultivars of industrial hemp (Xia et al., 2019). A collection of 5,838 proteins were quantified in Poplar plants to check up- or down-regulation of proteins that play a role in phytoremediation in solely “Cd stressed” and “Cd stress remediated with nitrogen” groups (Huang et al., 2020). In the study, the differentially expressed proteins were in the high double digits and hundreds. The following pathways were also upregulated (in the process category) in Cd + N (nitrogen) plants compared to the Cd only group; inositol metabolic process, polyol biosynthetic process, polyol metabolic process, alcohol biosynthetic process, monosaccharide metabolic process, hexose metabolic process, and phospholipid biosynthetic process showcasing that nitrogen has the potential to recover phyto-destructive events (Huang et al., 2020). Furthermore, in the same study, there was upregulation of the following candidate proteins at both the proteome and phosphoproteome levels: heat shock protein 70 (HSP70), 14–3–3 protein, peroxidase (POD), zinc finger protein (ZFP), ABC transporter protein, eukaryotic translation initiation factor (elF), and splicing factor 3 B subunit 1-like (SF3BI). In fact, plant transport and absorption were optimized, with 11 binding proteins, seven transporter proteins, and five-storage proteins upregulated in the Cd + N treatment. The main transporters that were upregulated were ABC transporters, which represented 57.1% of total transporters that were upregulated in the Cd + N treatment (Huang et al., 2020).

Biotechnologically, three main strategies are embarked upon to improve the clean-up of PTEs (i) manipulating metal/metalloid transporters, (ii) enhancing metal/metalloid ligand production, and (iii) conversion of metal/metalloid into volatile and less detrimental forms (Mosa et al., 2016). For the first strategy, tinkering with aquaporins that are capable of As transport, as well as other metalloids, antimonite (SbIII), silicon (Si), and boron (B) can be one way forward. The As is known to be present in rice grains and contributes to As in the human body (Chowdhury et al., 2020). For the second strategy, cysteine-rich proteins such as metallothionein and glutathione S-transferase take precedence, and this is a well-researched area in phytoremediation (Mosa et al., 2016). For the third one, Se, which is an essential micronutrient that can have negative repercussions when consumed in excess, is seen as a contender for intervention to turn excess Se into volatile products, such as dimethyl selenide, that can be released into the air (Mosa et al., 2016).

**Figure 4:**
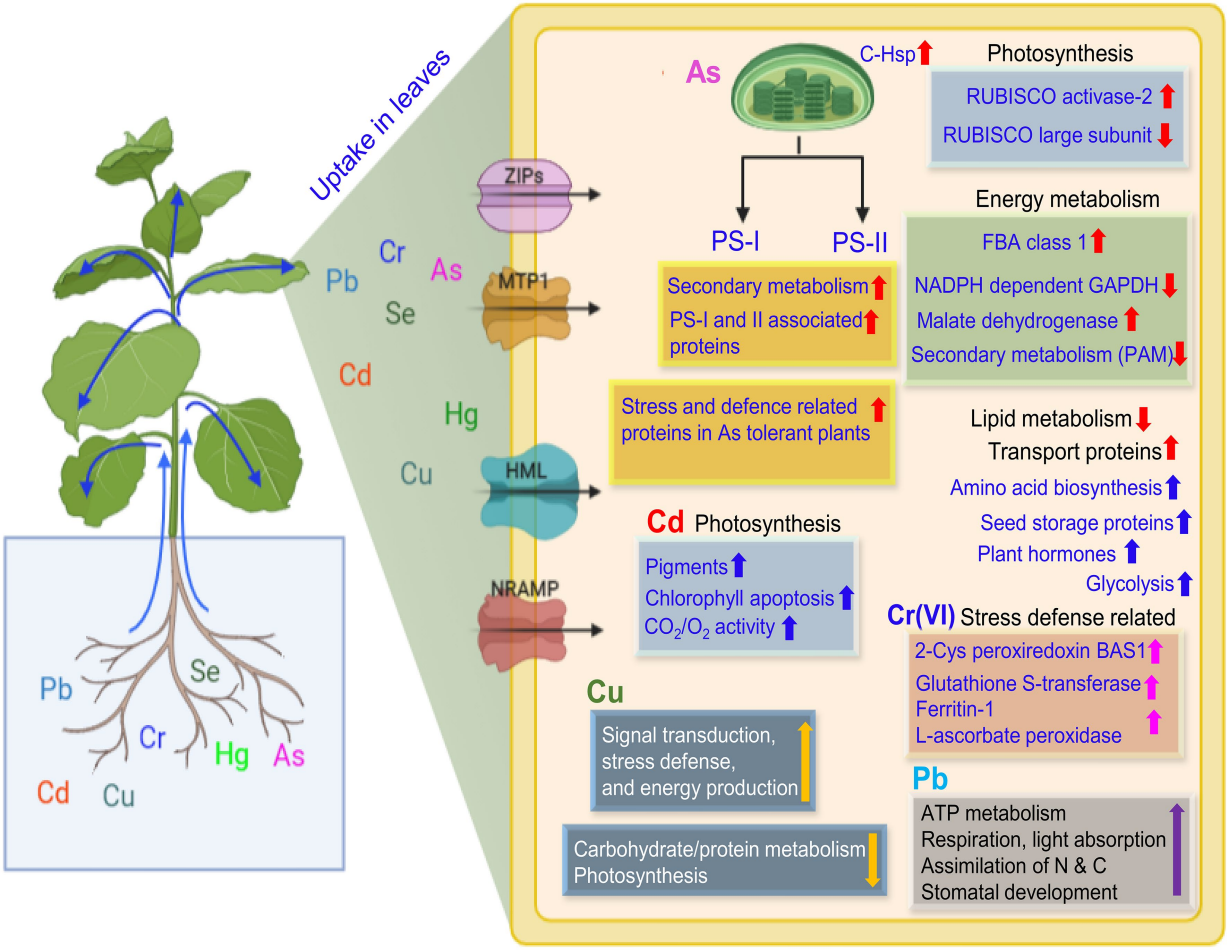
An illustration of the uptake of potentially toxic elements and plant response in the leaves. Uptake of PTEs in plant leaf resulting in significant up- or down-regulation of several proteins as represented by up and down arrows, respectively. The fold change may vary with the metal species, exposure duration, and intercellular concentration. Up and down red arrows are for as, blue for Cd, pink for Cr(VI), yellow for Cu, and violet for Pb. Membrane-embedded channels show the metal transport inside the leaf cell. Abbreviations: RUBISCO, Ribulose bisphosphate carboxylase/oxygenase; FBA, Ructose-bisphosphate aldolase; GAPDH, Glyceraldehyde 3-phosphate dehydrogenase; PAM, Phenylalanine aminomutase; C-Hsp, Chloroplast heat shock proteins; ZIPs, zinc-iron permease; MTP1, Metal transport protein1; CDF, Cation diffusion facilitator; and NRAMP, Natural resistant associated macrophage protein.

**Figure 5:**
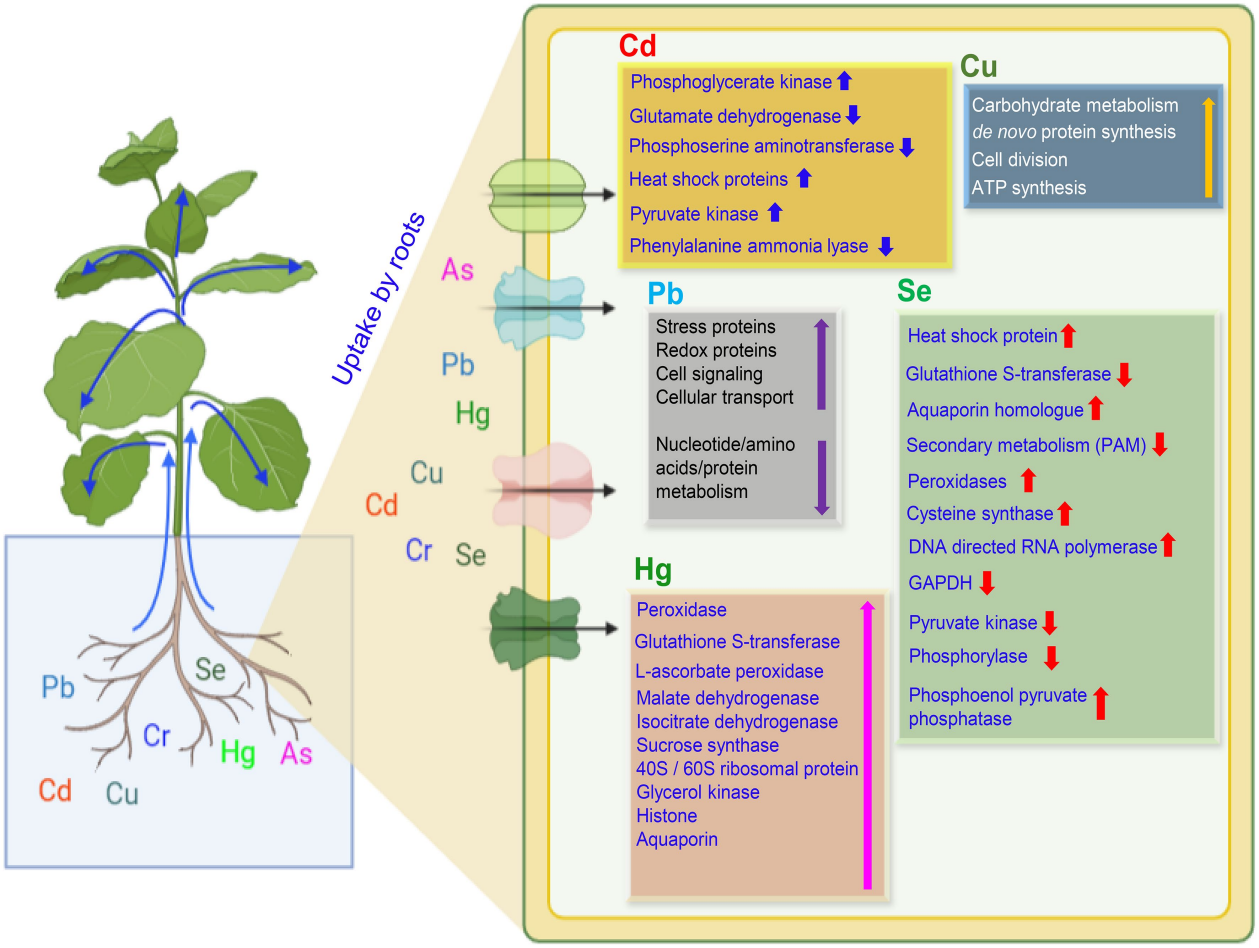
A proposed model on the uptake of potentially toxic elements and plant response in the roots. Uptake of PTEs by plant roots causing significant up- or down-regulation of essential proteins or enzymes as represented by up and down arrows, respectively. Up and down blue arrows are for Cd, yellow for Cu, violet for Pb, red for Se, and pink for Hg. Abbreviations: GAPDH, Glyceraldehyde 3-phosphate dehydrogenase, and ATP, Adenosine triphosphate.

Studies available to date report either the up- or down-regulation of a considerable number of proteins related to several cellular essential processes. A general observation cannot be made from these studies since the change in proteome profile may depend on many factors, including the type of metal, the concentration of metal, exposure duration, growth environment, and other biological or non-biological entities associated with the plant system. However, it can be suggested that the toxic outcome of PTEs lies in the profile of functional proteins subject to change by various parameters being major among them is the metal type/concentration. Some of the essential proteins/enzymes and their expression altered by PTEs in leaves and roots are presented in Figures 4, 5, respectively. Since there can be hundreds of proteins in a single type of plant tissue whose expression is changed by PTEs when comparative proteomics is performed, therefore, combining all under one umbrella is cumbersome. To understand the impact of specific PTE on a specific plant species, proteomic toxicity profiling of PTEs with respect to plant organs or tissue needs to be performed in future studies. Many hyperaccumulator species of Brassicaceae and Caryophyllaceae do not possess mycorrhizal networks in their roots. However, hyperaccumulator plants (for example, the genus *Thlaspi*) have been documented to possess mycorrhizae, although sparsely under field and experimental conditions (Ferrol et al., 2016). The inverse—mycorrhizae as determined by spore counts or root colonization has been significantly lower in soils rich in PTEs than non-metal rich soils—appears to claim that PTEs can have a detrimental effect on mycorrhizal survival (Ferrol et al., 2016).

However, mycorrhizal fungi never disappear from the ambient soil, suggesting that they can reform symbioses with plant roots under opportunistic conditions. AM fungi are capable of resisting PTEs by collective means that include cell wall binding to metals, chelation by glomalin, increased efflux to the exterior while diminishing uptake, cytosolic chelation, compartmentalization in the vacuoles, and upregulating antioxidant responses (Ferrol et al., 2016).

The success of exogenous nitrogen application suggests that the application of nitrogen-fixers (diazotrophs such as *Rhizobium* and *Azotobacter*) as biofertilizers can be an option to remediate polluted soils as well promote the capacity of plants to remediate PTEs such as Cd. In fact, metal-resisting Rhizobia can alleviate PTEs stress by production of phytohormones, nitrogen fixation, phosphorus solubilization, ACC deaminase synthesis, and siderophores (Brígido and Glick, 2015). The opulence in phenotypic and genotypic Rhizobial diversity makes it essential to choose the correct elite strains which can remediate soils that are not conducive to plant growth, such as N-deficient degraded lands.

## Conclusion

The PTEs accumulation in the environment above threshold levels poses a high risk to biota health and significantly undermines global environmental sustainability efforts. Phytoremediation has proven to be one of the most efficient strategies to remediate PTEs polluted sites. However, the large-scale application and commercial success of phytoremediation are still to be demonstrated, partly due to the limited understanding of the PTEs sequestration mechanisms. Although several successes were recorded, the evolution of plant proteomics provides further opportunity to sufficiently elucidate PTEs phytoremediation mechanisms, particularly in known high metal accumulating plants. This comprehensive review has demonstrated the potential of several PTEs accumulating plants and the active involvement of their proteome specific to the internal and/or external stimuli of applied PTEs. Various advanced tools and techniques both gel dependent and gel-free methods including qRT-PCR, western blotting, 2D-GE, LC-MS/MS, and MALDI TOF MS/MS have recognized the association of specific PTE with the enhanced expression of resulting proteome. The alteration of proteome expression (up- or down-regulation) in response to applied PTEs such as Cd, Cr, or Hg depends on the intracellular concentration of accumulated PTE, plant species, and the phytoremediation strategy being deployed by the plant. However, the PTE’s concentration effectively mitigated by the plant species in a defined or local environment without reducing crop production still needs further investigation. The species-specific (both plant and PTE’s species) knowledge of plant proteome changes under different growth conditions and growth phases such as from vegetation to flowering to fruiting stage indeed requires further investigation. A better understanding of PTEs-proteome relation will provide obvious benefits like (i) sustainable and effective decontamination of PTEs polluted sites while maintaining the plant growth and crop production and (ii) protection of soil biodiversity and quality. With an enhanced mechanistic understanding of the process, studies focusing on the engineering of the existing mechanisms of a plant’s PTEs sequestration should be prioritized. This will enable the development of an increased number of transgenic plant species with enhanced PTEs tolerance, uptake, and detoxification capabilities.

## Author Contributions

KU and MA: conceptualization. KU and BA: software. KU, MA, and BA: formal analysis. MA and HA: resources and funding acquisition. KU: writing—original draft preparation. KU, MA, HA, MR, MS, and BA: writing—review and editing. MA, KU, and HA: project administration. All authors contributed to the article and approved the submitted version.

## Funding

Qatar University’s Agricultural Research Station (ARS) supported this manuscript preparation and funded the APC.

## Conflict of Interest

The authors declare that the research was conducted in the absence of any commercial or financial relationships that could be construed as a potential conflict of interest.

The handling Editor declared a past collaboration with the author MS at the time of the review.

## Publisher’s Note

All claims expressed in this article are solely those of the authors and do not necessarily represent those of their affiliated organizations, or those of the publisher, the editors and the reviewers. Any product that may be evaluated in this article, or claim that may be made by its manufacturer, is not guaranteed or endorsed by the publisher.

## References

[ref1] Adiloğlu S. Açikgöz F. E. Gürgan M. (2021). Use of phytoremediation for pollution removal of hexavalent chromium-contaminated acid agricultural soils. Global. Nest 23, 400–406. doi: 10.30955/gnj.003433

[ref2] Ahmad P. Alam P. Balawi T. H. Altalayan F. H. Ahanger M. A. Ashraf M. (2020a). Sodium nitroprusside (SNP) improves tolerance to arsenic (As) toxicity in *Vicia faba* through the modifications of biochemical attributes, antioxidants, ascorbate-glutathione cycle and glyoxalase cycle. Chemosphere 244:125480. doi: 10.1016/j.chemosphere.2019.125480, PMID: 31821927

[ref3] Ahmad R. Ali S. Rizwan M. Dawood M. Farid M. Hussain A.. (2020b). Hydrogen sulfide alleviates chromium stress on cauliflower by restricting its uptake and enhancing antioxidative system. Physiol. Plant. 168, 289–300. doi: 10.1111/ppl.13001, PMID: 31209886

[ref4] Ahsan N. Renaut J. Komatsu S. (2009). Recent developments in the application of proteomics to the analysis of plant responses to heavy metals. Proteomics 9, 2602–2621. doi: 10.1002/pmic.200800935, PMID: 19405030

[ref5] Ali J. Mahmood T. Hayat K. Afridi M. S. Ali F. Chaudhary H. J. (2018). Phytoextraction of Cr by maize (*Zea mays* L.): the role of plant growth promoting endophyte and citric acid under polluted soil. Arch. Environ. Prot. 44, 73–82. doi: 10.24425/119705

[ref6] Alsafran M. Usman K. Al Jabri H. Rizwan M. (2021). Ecological and health risks assessment of potentially toxic metals and metalloids contaminants: A case study of agricultural soils in Qatar. Toxics 9:35. doi: 10.3390/toxics9020035, PMID: 33673174 PMC7918611

[ref7] Alsahli A. A. Bhat J. A. Alyemeni M. N. Ashraf M. Ahmad P. (2020). Hydrogen sulfide (H 2 S) mitigates arsenic (As)-induced toxicity in pea (*Pisum sativum* L.) plants by regulating osmoregulation, antioxidant defense system, Ascorbate glutathione cycle and Glyoxalase system. J. Plant Growth Regul. 1–17, 2515–2531. doi: 10.1007/s00344-020-10254-6

[ref8] Al-Thani R. F. Yasseen B. T. (2020). Phytoremediation of polluted soils and waters by native Qatari plants: future perspectives. Environ. Pollut. 259:113694. doi: 10.1016/j.envpol.2019.113694, PMID: 31887591

[ref9] Ariani A. Barozzi F. Sebastiani L. di Toppi L. S. di Sansebastiano G. Pietro A. (2019). AQUA1 is a mercury sensitive *poplar* aquaporin regulated at transcriptional and post-translational levels by Zn stress. Plant Physiol. Biochem. 135, 588–600. doi: 10.1016/j.plaphy.2018.10.038, PMID: 30424909

[ref10] Aweng E. R. Irfan A. H. M. Liyana A. A. Aisyah S. O. S. (2018). Potential of phytoremediation using *Scirpus validus* for domestic waste open dumping leachate. J. Appl. Sci. Environ. Manag. 22, 74–78. doi: 10.4314/jasem.v22i1.13

[ref11] Baig M. A. Ahmad J. Bagheri R. Ali A. A. Al-Huqail A. A. Ibrahim M. M.. (2018). Proteomic and ecophysiological responses of soybean (*Glycine max* L.) root nodules to Pb and hg stress. BMC Plant Biol. 18, 283–221. doi: 10.1186/s12870-018-1499-7, PMID: 30428829 PMC6237034

[ref12] Baldwin S. A. Khoshnoodi M. Rezadehbashi M. Taupp M. Hallam S. Mattes A.. (2015). The microbial community of a passive biochemical reactor treating arsenic, zinc, and sulfate-rich seepage. Front. Bioeng. Biotechnol. 3:27. doi: 10.3389/fbioe.2015.00027 25798439 PMC4351619

[ref13] Bezie Y. Taye M. Kumar A. (2021). “Recent advancement in phytoremediation for removal of toxic compounds,” in Nanobiotechnology for Green Environment, eds. Kumar A. Ram C. (United States: CRC Press), 195–228.

[ref14] Bhat J. A. Ahmad P. Corpas F. J. (2021). Main nitric oxide (NO) hallmarks to relieve arsenic stress in higher plants. J. Hazard. Mater. 406:124289. doi: 10.1016/j.jhazmat.2020.124289, PMID: 33153789

[ref15] Bisht R. Chanyal S. Srivastava R. K. (2020). A systematic review on phytoremediation technology: removal of pollutants from waste water and soil. Int J Res Eng Sci Manag. 3, 54–59.

[ref16] Brígido C. Glick B. R. (2015). “Phytoremediation using *rhizobia*,” in Phytoremediation eds. Ansari A. A. Gill S. S. Gill R. Lanz G. R. Newman Lee (United States: Springer), 95–114.

[ref17] Bukhari S. A. H. Zheng W. Xie L. Zhang G. Shang S. Wu F. (2016). Cr-induced changes in leaf protein profile, ultrastructure and photosynthetic traits in the two contrasting tobacco genotypes. Plant Growth Regul. 79, 147–156. doi: 10.1007/s10725-015-0120-4

[ref18] Cassidy L. Helbig A. O. Kaulich P. T. Weidenbach K. Schmitz R. A. Tholey A. (2021). Multidimensional separation schemes enhance the identification and molecular characterization of low molecular weight proteomes and short open reading frame-encoded peptides in top-down proteomics. J. Proteome 230:103988. doi: 10.1016/j.jprot.2020.103988, PMID: 32949814

[ref19] Chauhan R. Awasthi S. Indoliya Y. Chauhan A. S. Mishra S. Agrawal L.. (2020). Transcriptome and proteome analyses reveal selenium mediated amelioration of arsenic toxicity in rice (*Oryza sativa* L.). J. Hazard. Mater. 390:122122. doi: 10.1016/j.jhazmat.2020.122122, PMID: 32006842

[ref20] Chowdhary P. Yadav A. Singh R. Chandra R. Singh D. P. Raj A.. (2018). Stress response of *Triticum aestivum* L. and *Brassica juncea* L. against heavy metals growing at distillery and tannery wastewater contaminated site. Chemosphere 206, 122–131. doi: 10.1016/j.chemosphere.2018.04.156, PMID: 29738902

[ref21] Chowdhury N. R. Das A. Joardar M. De A. Mridha D. Das R.. (2020). Flow of arsenic between rice grain and water: its interaction, accumulation and distribution in different fractions of cooked rice. Sci. Total Environ. 731:138937. doi: 10.1016/j.scitotenv.2020.138937, PMID: 32402904

[ref22] Cvjetko P. Zovko M. Balen B. (2014). Proteomics of heavy metal toxicity in plants. Arch. Ind. Hyg. Toxicol. 65, 1–7. doi: 10.2478/10004-1254-65-2014-2443 24526604

[ref23] da Conceição Gomes M. A. Hauser-Davis R. A. de Souza A. N. Vitória A. P. (2016). Metal phytoremediation: general strategies, genetically modified plants and applications in metal nanoparticle contamination. Ecotoxicol. Environ. Saf. 134, 133–147. doi: 10.1016/j.ecoenv.2016.08.024 27611221

[ref24] De Bellis L. Aprile A. (2020). Heavy Metals Accumulation, Toxicity and Detoxification in Plants. Basel: MDPI-Multidisciplinary Digital Publishing Institute. eds. De Bellis L. Aprile A..

[ref25] de Castro Ribeiro P. R. C. Viana D. G. Pires F. R. Egreja Filho F. B. Bonomo R. Cargnelutti Filho A.. (2018). Selection of plants for phytoremediation of barium-polluted flooded soils. Chemosphere 206, 522–530. doi: 10.1016/j.chemosphere.2018.05.056, PMID: 29778077

[ref26] Dhir B. (2021). “Role of transporters of copper, manganese, zinc, and nickel in plants exposed to heavy metal stress,” in Metal and Nutrient Transporters in Abiotic Stress. eds. Roychoudhury A. Kunar Tripathi D. Deshmukh R. (United States: Elsevier), 145–168.

[ref27] Ding W. Zhang J. Wu S.-C. Zhang S. Christie P. Liang P. (2019). Responses of the grass *Paspalum distichum* L. to hg stress: A proteomic study. Ecotoxicol. Environ. Saf. 183:109549. doi: 10.1016/j.ecoenv.2019.109549, PMID: 31408818

[ref28] Dinu C. Vasile G.-G. Buleandra M. Popa D. E. Gheorghe S. Ungureanu E.-M. (2020). Translocation and accumulation of heavy metals in *Ocimum basilicum* L. plants grown in a mining-contaminated soil. J. Soils Sediments 20, 2141–2154. doi: 10.1007/s11368-019-02550-w

[ref29] Dixit G. Singh A. P. Kumar A. Dwivedi S. Deeba F. Kumar S.. (2015). Sulfur alleviates arsenic toxicity by reducing its accumulation and modulating proteome, amino acids and thiol metabolism in rice leaves. Sci. Rep. 5, 1–16. doi: 10.1038/srep16205 PMC4639781 26552588

[ref30] Domka A. Rozpądek P. Ważny R. Jędrzejczyk R. J. Hubalewska-Mazgaj M. Gonnelli C.. (2020). Transcriptome response of metallicolous and a non-metallicolous ecotypes of *noccaea goesingensis* to nickel excess. Plan. Theory 9:951. doi: 10.3390/plants9080951, PMID: 32731524 PMC7464472

[ref31] El-Amier Y. Elhindi K. El-Hendawy S. Al-Rashed S. Abd-ElGawad A. (2019). Antioxidant system and biomolecules alteration in Pisum sativum under heavy metal stress and possible alleviation by 5-aminolevulinic acid. Molecules 24:4194. doi: 10.3390/molecules24224194, PMID: 31752309 PMC6891517

[ref32] Fan K.-T. Rendahl A. K. Chen W.-P. Freund D. M. Gray W. M. Cohen J. D.. (2016). Proteome scale-protein turnover analysis using high resolution mass spectrometric data from stable-isotope labeled plants. J. Proteome Res. 15, 851–867. doi: 10.1021/acs.jproteome.5b00772, PMID: 26824330 PMC5482238

[ref33] Farooq M. A. Hong Z. Islam F. Noor Y. Hannan F. Zhang Y.. (2021). Comprehensive proteomic analysis of arsenic induced toxicity reveals the mechanism of multilevel coordination of efficient defense and energy metabolism in two *Brassica napus* cultivars. Ecotoxicol. Environ. Saf. 208:111744. doi: 10.1016/j.ecoenv.2020.111744, PMID: 33396070

[ref34] Ferrol N. Tamayo E. Vargas P. (2016). The heavy metal paradox in arbuscular mycorrhizas: from mechanisms to biotechnological applications. J. Exp. Bot. 67, 6253–6265. doi: 10.1093/jxb/erw403 27799283

[ref35] Gautam V. Sharma P. Bakshi P. Arora S. Bhardwaj R. Paray B. A.. (2020). Effect of *Rhododendron arboreum* leaf extract on the antioxidant defense system against chromium (VI) stress in *Vigna radiata* plants. Plan. Theory 9:164. doi: 10.3390/plants9020164, PMID: 32013242 PMC7076638

[ref36] Gill R. A. Ahmar S. Ali B. Saleem M. H. Khan M. U. Zhou W.. (2021). The role of membrane transporters in plant growth and development, and abiotic stress tolerance. Int. J. Mol. Sci. 22:12792. doi: 10.3390/ijms222312792, PMID: 34884597 PMC8657488

[ref37] Goodin M. M. (2018). Protein localization and interaction studies in plants: toward defining complete proteomes by visualization. Adv. Virus Res. 100, 117–144. doi: 10.1016/bs.aivir.2017.10.004, PMID: 29551133

[ref38] Gutsch A. Sergeant K. Keunen E. Prinsen E. Guerriero G. Renaut J.. (2019a). Does long-term cadmium exposure influence the composition of pectic polysaccharides in the cell wall of *Medicago sativa* stems? BMC Plant Biol. 19, 1–17. doi: 10.1186/s12870-019-1859-y 31226937 PMC6588869

[ref39] Gutsch A. Sergeant K. Renaut J. (2019b). Application of bottom-up and top-down proteomics in Medicago spp. Model Legum. *Medicago truncatula*, ed. de Bruijn F., 1087–1095. doi: 10.1002/9781119409144.ch141

[ref40] Habiba U. Ali S. Rizwan M. Ibrahim M. Hussain A. Shahid M. R.. (2019). Alleviative role of exogenously applied mannitol in maize cultivars differing in chromium stress tolerance. Environ. Sci. Pollut. Res. 26, 5111–5121. doi: 10.1007/s11356-018-3970-2, PMID: 30607836

[ref41] Hamidpour M. Nemati H. Dahaji P. A. Roosta H. R. (2020). Effects of plant growth-promoting bacteria on EDTA-assisted phytostabilization of heavy metals in a contaminated calcareous soil. Environ. Geochem. Health 42, 2535–2545. doi: 10.1007/s10653-019-00422-3, PMID: 31583504

[ref42] Hasanuzzaman M. Nahar K. Rahman A. Mahmud J. Al Alharby H. F. Fujita M., (2018). Exogenous glutathione attenuates lead-induced oxidative stress in wheat by improving antioxidant defense and physiological mechanisms. J. Plant Interact. 13, 203–212. doi: 10.1080/17429145.2018.1458913

[ref43] Hasegawa H. Rahman I. M. M. Rahman M. A., (2016). Environmental Remediation Technologies for Metal-Contaminated Soils. (United States: Springer).

[ref44] He Y. Langenhoff A. A. Sutton N. B. Rijnaarts H. H. Blokland M. H. Chen F.. (2017). Metabolism of ibuprofen by *Phragmites australis*: uptake and phytodegradation. Environ. Sci. Technol. 51, 4576–4584. doi: 10.1021/acs.est.7b00458, PMID: 28346781 PMC5770141

[ref45] Hego E. Vilain S. Barré A. Claverol S. Dupuy J. Lalanne C.. (2016). Copper stress-induced changes in leaf soluble proteome of cu-sensitive and tolerant *Agrostis capillaris* L. populations. Proteomics 16, 1386–1397. doi: 10.1002/pmic.201500083, PMID: 26900021

[ref46] Hossain Z. Komatsu S. (2013). Contribution of proteomic studies towards understanding plant heavy metal stress response. Front. Plant Sci. 3:310. doi: 10.3389/fpls.2012.00310 23355841 PMC3555118

[ref47] Huang J. Wu X. Tian F. Chen Q. Luo P. Zhang F.. (2020). Changes in proteome and protein phosphorylation reveal the protective roles of exogenous nitrogen in alleviating cadmium toxicity in poplar plants. Int. J. Mol. Sci. 21:278. doi: 10.3390/ijms21010278 PMC6982014 31906144

[ref48] Jeyasundar P. G. S. A. Ali A. Azeem M. Li Y. Guo D. Sikdar A.. (2021). Green remediation of toxic metals contaminated mining soil using bacterial consortium and *Brassica juncea*. Environ. Pollut. 277:116789. doi: 10.1016/j.envpol.2021.116789, PMID: 33640810

[ref49] Jogawat A. Yadav B. Narayan O. P. (2021). Metal transporters in organelles and their roles in heavy metal transportation and sequestration mechanisms in plants. Physiol. Plant 173, 259–275. doi: 10.1111/ppl.13370 33586164

[ref50] Jorrin-Novo J. V. Komatsu S. Sanchez-Lucas R. de Francisco L. E. R. (2019). Gel electrophoresis-based plant proteomics: past, present, and future. Happy 10th anniversary journal of proteomics! J. Proteome 198, 1–10. doi: 10.1016/j.jprot.2018.08.016, PMID: 30170112

[ref51] Kang G. Li G. Wang L. Wei L. Yang Y. Wang P.. (2015). Hg-responsive proteins identified in wheat seedlings using iTRAQ analysis and the role of ABA in hg stress. J. Proteome Res. 14, 249–267. doi: 10.1021/pr5006873, PMID: 25330896

[ref52] Karasinski J. Wrobel K. Corrales Escobosa A. R. Konopka A. Bulska E. Wrobel K. (2017). *Allium cepa* L. response to sodium selenite (se (IV)) studied in plant roots by a LC-MS-based proteomic approach. J. Agric. Food Chem. 65, 3995–4004. doi: 10.1021/acs.jafc.7b01085, PMID: 28467079

[ref53] Kaszycki P. Dubicka-Lisowska A. Augustynowicz J. Piwowarczyk B. Wesołowski W. (2018). *Callitriche cophocarpa* (water starwort) proteome under chromate stress: evidence for induction of a quinone reductase. Environ. Sci. Pollut. Res. 25, 8928–8942. doi: 10.1007/s11356-017-1067-y, PMID: 29332274 PMC5854755

[ref54] Klimenko O. Pernis M. Danchenko M. Skultéty L. Klubicová K. Shevchenko G. (2019). Natural ecotype of *Arabidopsis thaliana* (L.) Heynh (Chernobyl-07) respond to cadmium stress more intensively than the sensitive ecotypes oasis and Columbia. Ecotoxicol. Environ. Saf. 173, 86–95. doi: 10.1016/j.ecoenv.2019.02.012, PMID: 30769207

[ref55] Kosakivska I. V. Babenko L. M. Romanenko K. O. Korotka I. Y. Potters G. (2021). Molecular mechanisms of plant adaptive responses to heavy metals stress. Cell Biol. Int. 45, 258–272. doi: 10.1002/cbin.11503, PMID: 33200493

[ref56] Kosová K. Vítámvás P. Urban M. O. Prášil I. T. Renaut J. (2018). Plant abiotic stress proteomics: the major factors determining alterations in cellular proteome. Front. Plant Sci. 9:122. doi: 10.3389/fpls.2018.00122, PMID: 29472941 PMC5810178

[ref57] Kumar A. Prasad M. N. V. (2018). Plant-lead interactions: transport, toxicity, tolerance, and detoxification mechanisms. Ecotoxicol. Environ. Saf. 166, 401–418. doi: 10.1016/j.ecoenv.2018.09.113, PMID: 30290327

[ref58] Kumar V. Singh J. Chopra A. K. (2018). Assessment of plant growth attributes, bioaccumulation, enrichment, and translocation of heavy metals in water lettuce (*Pistia stratiotes* L.) grown in sugar mill effluent. Int. J. Phytoremediation 20, 507–521. doi: 10.1080/15226514.2017.1393391, PMID: 29608378

[ref59] Kumar B. Smita K. Flores L. C. (2017). Plant mediated detoxification of mercury and lead. Arab. J. Chem. 10, S2335–S2342. doi: 10.1016/j.arabjc.2013.08.010

[ref60] Kumari A. Pandey-Rai S. (2018). Enhanced arsenic tolerance and secondary metabolism by modulation of gene expression and proteome profile in *Artemisia annua* L. after application of exogenous salicylic acid. Plant Physiol. Biochem. 132, 590–602. doi: 10.1016/j.plaphy.2018.10.010, PMID: 30326438

[ref61] Lai J. Zhang-Xuan D. Xiao-Hui J. I. Xue-Gang L. (2020). Absorption and interaction mechanisms of uranium & cadmium in purple sweet potato (*Ipomoea batatas* L.). J. Hazard. Mater. 400:123264. doi: 10.1016/j.jhazmat.2020.123264, PMID: 32947695

[ref62] Lan X.-Y. Yan Y.-Y. Yang B. Li X.-Y. Xu F.-L. (2018). Differential expression of proteins in the leaves and roots of cadmium-stressed *Microsorum pteropus*, a novel potential aquatic cadmium hyperaccumulator. Sci. Total Environ. 642, 1369–1377. doi: 10.1016/j.scitotenv.2018.06.168, PMID: 30045517

[ref63] Li G. K. Gao J. Peng H. Shen Y. O. Ding H. P. Zhang Z. M.. (2016). Proteomic changes in maize as a response to heavy metal (lead) stress revealed by iTRAQ quantitative proteomics. Genet. Mol. Res. 15, 1–14. doi: 10.4238/gmr.15017254 26909923

[ref64] Li G. Peng X. Xuan H. Wei L. Yang Y. Guo T.. (2013). Proteomic analysis of leaves and roots of common wheat (*Triticum aestivum* L.) under copper-stress conditions. J. Proteome Res. 12, 4846–4861. doi: 10.1021/pr4008283, PMID: 24074260

[ref65] Liu Y. Damaris R. N. Yang P. (2017). Proteomics analysis identified a DRT protein involved in arsenic resistance in *Populus*. Plant Cell Rep. 36, 1855–1869. doi: 10.1007/s00299-017-2199-8, PMID: 28815368

[ref66] Ludvíková M. Griga M. (2019). “Transgenic fiber crops for phytoremediation of metals and metalloids” in Transgenic Plant Technology for Remediation of Toxic Metals and Metalloids, eds. Vara Prasad M. N. (United States: Elsevier), 341–358.

[ref67] Luo J.-S. Zhang Z. (2019). Proteomic changes in the xylem sap of Brassica napus under cadmium stress and functional validation. BMC Plant Biol. 19, 1–14. doi: 10.1186/s12870-019-1895-7 31242871 PMC6595625

[ref68] Maestri E. Marmiroli M. Visioli G. Marmiroli N. (2010). Metal tolerance and hyperaccumulation: costs and trade-offs between traits and environment. Environ. Exp. Bot. 68, 1–13. doi: 10.1016/j.envexpbot.2009.10.011

[ref69] Mahajan P. Kaushal J. (2018). Role of phytoremediation in reducing cadmium toxicity in soil and water. J. Toxicol. 2018, 1–16. doi: 10.1155/2018/4864365, PMID: 30425738 PMC6218723

[ref70] Meena K. K. Sorty A. M. Bitla U. M. Choudhary K. Gupta P. Pareek A.. (2017). Abiotic stress responses and microbe-mediated mitigation in plants: the omics strategies. Front. Plant Sci. 8:172. doi: 10.3389/fpls.2017.00172 28232845 PMC5299014

[ref71] Meitei M. D. Prasad M. N. V. (2021). Potential of *Typha latifolia* L. for phytofiltration of iron-contaminated waters in laboratory-scale constructed microcosm conditions. Appl. Water Sci. 11, 1–10. doi: 10.1007/s13201-020-01339-4

[ref72] Meng D. Xu P. Dong Q. Wang S. Wang Z. (2017). Comparison of foliar and root application of potassium dihydrogen phosphate in regulating cadmium translocation and accumulation in tall fescue (*Festuca arundinacea*). Water Air Soil Pollut. 228, 118. doi: 10.1007/s11270-017-3304-x

[ref73] Mishra A. Mishra S. P. Arshi A. Agarwal A. Dwivedi S. K. (2020). “Plant-microbe interactions for bioremediation and phytoremediation of environmental pollutants and agro-ecosystem development,” in Bioremediation of Industrial Waste for Environmental Safety. eds. Saxena G. Naresh R. Bharagava (United States: Springer), 415–436.

[ref74] Mosa K. A. Saadoun I. Kumar K. Helmy M. Dhankher O. P. (2016). Potential biotechnological strategies for the cleanup of heavy metals and metalloids. Front. Plant Sci. 7:303. doi: 10.3389/fpls.2016.00303 27014323 PMC4791364

[ref75] Najeeb U. Ahmad W. Zia M. H. Zaffar M. Zhou W. (2017). Enhancing the lead phytostabilization in wetland plant *Juncus effusus* L. through somaclonal manipulation and EDTA enrichment. Arab. J. Chem. 10, S3310–S3317. doi: 10.1016/j.arabjc.2014.01.009

[ref76] Nikolić M. Stevović S. (2015). Family Asteraceae as a sustainable planning tool in phytoremediation and its relevance in urban areas. Urban For. Urban Green. 14, 782–789. doi: 10.1016/j.ufug.2015.08.002

[ref77] Paape T. Hatakeyama M. Shimizu-Inatsugi R. Cereghetti T. Onda Y. Kenta T.. (2016). Conserved but attenuated parental gene expression in allopolyploids: constitutive zinc hyperaccumulation in the allotetraploid *Arabidopsis kamchatica*. Mol. Biol. Evol. 33, 2781–2800. doi: 10.1093/molbev/msw141, PMID: 27413047 PMC5062318

[ref78] Pang X.-Q. Jia C.-Z. Wang W.-Y. (2015). Petroleum geology features and research developments of hydrocarbon accumulation in deep petroliferous basins. Pet. Sci. 12, 1–53. doi: 10.1007/s12182-015-0014-0

[ref79] Paunov M. Koleva L. Vassilev A. Vangronsveld J. Goltsev V. (2018). Effects of different metals on photosynthesis: cadmium and zinc affect chlorophyll fluorescence in durum wheat. Int. J. Mol. Sci. 19:787. doi: 10.3390/ijms19030787, PMID: 29522461 PMC5877648

[ref80] Peco J. D. Campos J. A. Romero-Puertas M. C. Olmedilla A. Higueras P. Sandalio L. M. (2020). Characterization of mechanisms involved in tolerance and accumulation of cd in *Biscutella auriculata* L. Ecotoxicol. Environ. Saf. 201:110784. doi: 10.1016/j.ecoenv.2020.110784, PMID: 32485494

[ref81] Pidatala V. R. Li K. Sarkar D. Wusirika R. Datta R. (2018). Comparative metabolic profiling of vetiver (*Chrysopogon zizanioides*) and maize (*Zea mays*) under lead stress. Chemosphere 193, 903–911. doi: 10.1016/j.chemosphere.2017.11.087, PMID: 29874765

[ref82] Radziemska M. (2018). Study of applying naturally occurring mineral sorbents of Poland (dolomite halloysite, chalcedonite) for aided phytostabilization of soil polluted with heavy metals. Catena 163, 123–129. doi: 10.1016/j.catena.2017.12.015

[ref83] Rascio N. Navari-Izzo F. (2011). Heavy metal hyperaccumulating plants: how and why do they do it? And what makes them so interesting? Plant Sci. 180, 169–181. doi: 10.1016/j.plantsci.2010.08.016, PMID: 21421358

[ref84] Raza A. Habib M. Kakavand S. N. Zahid Z. Zahra N. Sharif R.. (2020). Phytoremediation of cadmium: physiological, biochemical, and molecular mechanisms. Biology 9:177. doi: 10.3390/biology9070177 PMC7407403 32708065

[ref85] Raza A. Razzaq A. Mehmood S. S. Hussain M. A. Wei S. He H.. (2021). Omics: The way forward to enhance abiotic stress tolerance in *Brassica napus* L. GM Crops Food 12, 251–281. doi: 10.1080/21645698.2020.1859898, PMID: 33464960 PMC7833762

[ref86] Reimegård J. Tarbier M. Danielsson M. Schuster J. Baskaran S. Panagiotou S.. (2021). A combined approach for single-cell mRNA and intracellular protein expression analysis. Commun. Biol. 4, 1–11. doi: 10.1038/s42003-021-02142-w 34035432 PMC8149646

[ref87] Rizvi A. Ahmed B. Zaidi A. Khan M. S. M. S. (2019). Heavy metal mediated phytotoxic impact on winter wheat: oxidative stress and microbial management of toxicity by: *Bacillus subtilis* BM2. RSC Adv. 9, 6125–6142. doi: 10.1039/c9ra00333a 35517307 PMC9060871

[ref88] Rizvi A. Zaidi A. Ameen F. Ahmed B. AlKahtani M. D. F. Khan M. S. (2020). Heavy metal induced stress on wheat: phytotoxicity and microbiological management. RSC Adv. 10, 38379–38403. doi: 10.1039/D0RA05610C PMC9121104 35693041

[ref89] Roy S. K. Cho S.-W. Kwon S. J. Kamal A. H. M. Kim S.-W. Oh M.-W.. (2016). Morpho-physiological and proteome level responses to cadmium stress in sorghum. PLoS One 11:e0150431. doi: 10.1371/journal.pone.0150431, PMID: 26919231 PMC4769174

[ref90] Sakakibara M. Watanabe A. Inoue M. Sano S. Kaise T. (2010). “Phytoextraction and phytovolatilization of arsenic from As-contaminated soils by Pteris vittata”, in: Proceedings of the annual international conference on soils, sediments, water and energy), 26.

[ref91] Sako A. Kandakar J. Tamari N. Higa A. Yamaguchi K. Kitamura Y. (2016). Copper excess promotes propagation and induces proteomic change in root cultures of *Hyoscyamus albus* L. Plant Physiol. Biochem. 103, 1–9. doi: 10.1016/j.plaphy.2016.02.032, PMID: 26945770

[ref92] Saleem M. H. Ali S. Rehman M. Hasanuzzaman M. Rizwan M. Irshad S.. (2020a). Jute: A potential candidate for phytoremediation of metals—A review. Plan. Theory 9:258. doi: 10.3390/plants9020258 PMC7076354 32079368

[ref93] Saleem M. H. Ali S. Rehman M. Rizwan M. Kamran M. Mohamed I. A.. (2020b). Individual and combined application of EDTA and citric acid assisted phytoextraction of copper using jute (*Corchorus capsularis* L.) seedlings. Environ. Tech. Innov. 19:100895. doi: 10.1016/j.eti.2020.100895

[ref94] Shaheen S. M. Rinklebe J. (2015). Phytoextraction of potentially toxic elements by Indian mustard, rapeseed, and sunflower from a contaminated riparian soil. Environ. Geochem. Health 37, 953–967. doi: 10.1007/s10653-015-9718-8, PMID: 26040974

[ref95] Shamim S. (2018). Biosorption of heavy metals. Biosorption 2, 21–49. doi: 10.5772/intechopen.72099

[ref96] Shankar S. (2017). “Management and remediation of problem soils, solid waste and soil pollution,” in Principles and Applications of Environmental Biotechnology for a Sustainable Future. ed. Lakhan Singh R. (United States: Springer), 143–171.

[ref97] Sharma P. Pandey S. (2014). Status of phytoremediation in world scenario. Int. J. Environ. Bioremediation Biodegrad. 2, 178–191. doi: 10.12691/ijebb-2-4-5

[ref98] Sidoli S. Lu C. Coradin M. Wang X. Karch K. R. Ruminowicz C.. (2017). Metabolic labeling in middle-down proteomics allows for investigation of the dynamics of the histone code. Epigenetics Chromatin 10, 1–15. doi: 10.1186/s13072-017-0139-z 28683815 PMC5501349

[ref99] Singh S. Fulzele D. P. (2021). Phytoextraction of arsenic using a weed plant Calotropis procera from contaminated water and soil: growth and biochemical response. Int. J. Phytoremediation 23, 1310–1318. doi: 10.1080/15226514.2021.1895717 33725458

[ref100] Su N. Wu Q. Chen H. Huang Y. Zhu Z. Chen Y.. (2019). Hydrogen gas alleviates toxic effects of cadmium in *Brassica campestris* seedlings through up-regulation of the antioxidant capacities: possible involvement of nitric oxide. Environ. Pollut. 251, 45–55. doi: 10.1016/j.envpol.2019.03.094, PMID: 31071632

[ref101] Suman J. Uhlik O. Viktorova J. Macek T. (2018). Phytoextraction of heavy metals: a promising tool for clean-up of polluted environment? Front. Plant Sci. 9:1476. doi: 10.3389/fpls.2018.01476, PMID: 30459775 PMC6232834

[ref102] Surucu A. Marif A. A. Majid S. N. Farooq S. Tahir N. A.-R. (2020). Effect of different water sources and water availability regimes on heavy metal accumulation in two sunflower species. Carpathian J. Earth Environ. Sci. 15, 289–300. doi: 10.26471/cjees/2020/015/129

[ref103] Sytar O. Ghosh S. Malinska H. Zivcak M. Brestic M. (2021). Physiological and molecular mechanisms of metal accumulation in hyperaccumulator plants. Physiol. Plant. 173, 148–166. doi: 10.1111/ppl.13285, PMID: 33219524

[ref104] Szuba A. Lorenc-Plucińska G. (2018). Field proteomics of *Populus alba* grown in a heavily modified environment–An example of a tannery waste landfill. Sci. Total Environ. 610-611, 1557–1571. doi: 10.1016/j.scitotenv.2017.06.102 28712470

[ref105] Tian M. Yang Y. Ávila F. W. Fish T. Yuan H. Hui M.. (2018). Effects of selenium supplementation on glucosinolate biosynthesis in broccoli. J. Agric. Food Chem. 66, 8036–8044. doi: 10.1021/acs.jafc.8b03396, PMID: 29975053

[ref106] Usman K. Abu-Dieyeh M. H. Al-Ghouti M. A. (2019a). Evaluating the invasive plant, Prosopis juliflora in the two initial growth stages as a potential candidate for heavy metal phytostabilization in metalliferous soil. Environ. Pollut. Bioavailab. 31, 145–155. doi: 10.1080/26395940.2019.1585958

[ref107] Usman K. Abu-Dieyeh M. H. Zouari N. Al-Ghouti M. A. (2020a). Lead (Pb) bioaccumulation and antioxidative responses in Tetraena qataranse. Sci. Rep. 10, 1–10. doi: 10.1038/s41598-020-73621-z 33051495 PMC7555492

[ref108] Usman K. Al Jabri H. Abu-Dieyeh M. H. Alsafran M. H. S. A. (2020b). Comparative assessment of toxic metals bioaccumulation and the mechanisms of chromium (Cr) tolerance and uptake in *Calotropis procera*. Front. Plant Sci. 11:883. doi: 10.3389/fpls.2020.00883, PMID: 32636868 PMC7317033

[ref109] Usman K. Al-Ghouti M. A. Abu-Dieyeh M. H. (2018). Phytoremediation: halophytes as promising heavy metal hyperaccumulators. Heavy Met. 27:7378. doi: 10.5772/intechopen.73879

[ref110] Usman K. Al-Ghouti M. A. Abu-Dieyeh M. H. (2019b). The assessment of cadmium, chromium, copper, and nickel tolerance and bioaccumulation by shrub plant *Tetraena qataranse*. Sci. Rep. 9, 1–11. doi: 10.1038/s41598-019-42029-9 30948781 PMC6449511

[ref111] Viana D. G. Egreja Filho F. B. Pires F. R. Soares M. B. Ferreira A. D. Bonomo R.. (2021). In situ barium phytoremediation in flooded soil using Typha domingensis under different planting densities. Ecotoxicol. Environ. Saf. 210:111890. doi: 10.1016/j.ecoenv.2021.111890, PMID: 33440270

[ref112] Visioli G. Marmiroli N. (2013). The proteomics of heavy metal hyperaccumulation by plants. J. Proteome 79, 133–145. doi: 10.1016/j.jprot.2012.12.006, PMID: 23268120

[ref113] Wei F. Shahid M. J. Alnusairi G. S. H. Afzal M. Khan A. El-Esawi M. A.. (2020). Implementation of floating treatment wetlands for textile wastewater management: A review. Sustain. For. 12:5801. doi: 10.3390/su12145801

[ref114] Wei Z. Van Le Q. Peng W. Yang Y. Yang H. Gu H.. (2021). A review on phytoremediation of contaminants in air, water and soil. J. Hazard. Mater. 403:123658. doi: 10.1016/j.jhazmat.2020.123658 33264867

[ref115] Wiszniewska A. (2021). Priming strategies for benefiting plant performance under toxic trace metal exposure. Plan. Theory 10:623. doi: 10.3390/plants10040623, PMID: 33805922 PMC8064369

[ref116] Wuana R. A. Okieimen F. E., (2011). Heavy metals in contaminated soils: a review of sources, chemistry, risks and best available strategies for remediation. Int. Sch. Res. Not. 2011:20.

[ref117] Xia C. Hong L. Yang Y. Yanping X. Xing H. Gang D. (2019). Protein changes in response to lead stress of lead-tolerant and lead-sensitive industrial hemp using swath technology. Genes (Basel) 10:396. doi: 10.3390/genes10050396, PMID: 31121980 PMC6562531

[ref118] Xie X. He Z. Chen N. Tang Z. Wang Q. Cai Y. (2019). The roles of environmental factors in regulation of oxidative stress in plant. Biomed Res. Int. 2019:11. doi: 10.1155/2019/9732325 PMC6530150 31205950

[ref119] Yadav B. K. Siebel M. A. van Bruggen J. J. (2011). Rhizofiltration of a heavy metal (lead) containing wastewater using the wetland plant *Carex pendula*. *CLEAN–soil, air*. WaterSA 39, 467–474. doi: 10.1002/clen.201000385

[ref120] Yan A. Wang Y. Tan S. N. Mohd Yusof M. L. Ghosh S. Chen Z. (2020). Phytoremediation: a promising approach for revegetation of heavy metal-polluted land. Front. Plant Sci. 11:359. doi: 10.3389/fpls.2020.00359, PMID: 32425957 PMC7203417

[ref121] Yıldız M. Terzi H. (2016). Proteomic analysis of chromium stress and sulfur deficiency responses in leaves of two canola (*Brassica napus* L.) cultivars differing in Cr (VI) tolerance. Ecotoxicol. Environ. Saf. 124, 255–266. doi: 10.1016/j.ecoenv.2015.10.023, PMID: 26546907

[ref122] Zhang C. Xu B. Geng W. Shen Y. Xuan D. Lai Q.. (2019). Comparative proteomic analysis of pepper (*Capsicum annuum* L.) seedlings under selenium stress. PeerJ 7:e8020. doi: 10.7717/peerj.8020, PMID: 31799069 PMC6884995

[ref123] Zhang H. Zhang L.-L. Li J. Chen M. An R.-D. (2020). Comparative study on the bioaccumulation of lead, cadmium and nickel and their toxic effects on the growth and enzyme defence strategies of a heavy metal accumulator, *Hydrilla verticillata* (lf) Royle. Environ. Sci. Pollut. Res., 1–13, 9853–9865. 10.1007/s11356-019-06968-0 31927739

